# The genetic variation and relationship among the natural hybrids of *Mangifera casturi* Kosterm

**DOI:** 10.1038/s41598-021-99381-y

**Published:** 2021-10-05

**Authors:** Deden Derajat Matra, Muh Agust Nur Fathoni, Muhammad Majiidu, Hanif Wicaksono, Agung Sriyono, Gunawan Gunawan, Hilda Susanti, Rismita Sari, Fitmawati Fitmawati, Iskandar Zulkarnaen Siregar, Winarso Drajad Widodo, Roedhy Poerwanto

**Affiliations:** 1 Department of Agronomy and Horticulture, Faculty of Agriculture, Bogor Agricultural University (IPB University), Bogor, Indonesia; 2 Molecular Science Research Group, Advanced Research Laboratory, Bogor Agricultural University (IPB University), Bogor, Indonesia; 3 Tunas Meratus Conservation Organization of South Kalimantan, Kandangan, Indonesia; 4 Banua Botanical Garden, Province of South Kalimantan, Banjarbaru, Indonesia; 5 Department of Biology, Faculty of Mathematics and Natural Sciences, Lambung Mangkurat University, Banjarbaru, Indonesia; 6 Department of Agronomy, Faculty of Agriculture, Lambung Mangkurat University, Banjarbaru, Indonesia; 7 Research Centre for Plant Conservation and Botanic Gardens—Indonesian Institute of Sciences (LIPI), Bogor, Indonesia; 8 Department of Biology, Faculty of Mathematics and Natural Sciences, Riau University, Pekanbaru, Indonesia; 9 Department of Silviculture, Faculty of Forestry and Environment, Bogor Agricultural University (IPB University), Bogor, Indonesia

**Keywords:** Plant hybridization, Plant domestication, Phylogenomics, Plant molecular biology

## Abstract

*Mangifera casturi* Kosterm., a mango plant from Kalimantan Selatan, Indonesia, has limited genetic information, severely limiting the research on its genetic variation and phylogeny. We collected *M. casturi*’s genomic information using next-generation sequencing, developed microsatellite markers and performed Sanger sequencing for DNA barcoding analysis. These markers were used to confirm parental origin and genetic diversity of *M. casturi* hybrids. The clean reads of the Kasturi accession were assembled de novo, producing 259 872 scaffolds (N50 = 1 445 bp). Fourteen polymorphic microsatellite markers were developed from 11 040 microsatellite motif-containing sequences. In total, 58 alleles were produced with a mean of 4.14 alleles per locus. Microsatellite marker analysis revealed broad genetic variation in *M. casturi*. Phylogenetic analysis was performed using internal transcribed spacers (ITS), *matK*, *rbcL*, and *trnH-psbA*. The phylogenetic tree of chloroplast markers placed Kasturi, Cuban, Pelipisan, Pinari, and Hambawang in one group, with *M. indica* as the female ancestor. Meanwhile, the phylogenetic tree of ITS markers indicated several *Mangifera* species as ancestors of *M. casturi*. Thus, *M. casturi* very likely originated from the cross-hybridization of multiple ancestors. Furthermore, crossing the F1 hybrids of *M. indica* and *M. quadrifida* with other *Mangifera* spp. may have generated much genetic variation. The genetic information for *M. casturi* will be a resource for breeding improvement, and conservation studies.

## Introduction

**Figure 1:**
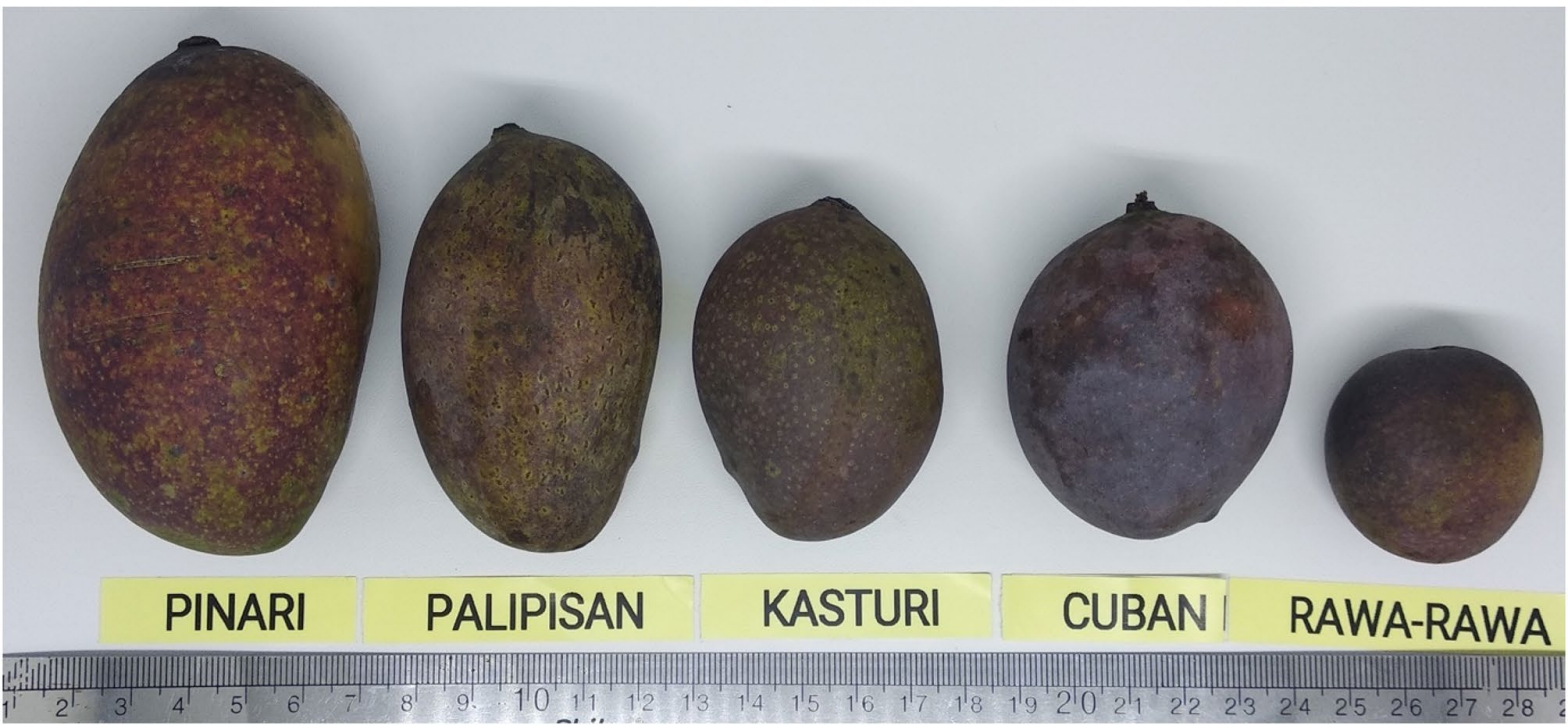
Appearance Fruits of *M. casturi* hybrids (from left to right: Pinari, Pelipisan, Kasturi, and Cuban) and closely related species (Rawa-rawa). This photo was taken by DDM.

*Mangifera casturi* Kosterm., or Kalimantan mango, is an endogenous fruit plant in Kalimantan Selatan, Indonesia; it is classified as extinct in the wild according to the IUCN Red List^1^. *M. casturi* belongs to the *Mangifera* genus within the Anacardiaceae family^2^ and is classified as a common ancestor of the *Mangifera* species in Indonesia^3^. *M. casturi* is proposed to be the natural hybrid of *M. indica* and *M. quadrifida* according to single nucleotide polymorphisms (SNPs) analysis^4^. In Kalimantan Selatan, *M. casturi* are known by various local names, such as Kasturi, Cuban, Pelipisan, Pinari, and Rawa-rawa (Fig. 1)^2^. *M. casturi* bears small fruits, with an attractive purple color and a distinctive aroma; thus, it is a prospective genetic resource for improving mango varieties in the future^5^. *M. casturi* also contains useful secondary metabolites, such as lupeol, an antioxidant and anticancer agent^6^. However, the genomic information on *M. casturi* remains limited, with only one accession deposited (MF678493.1) in nucleotide repositories such as the NCBI, and one SRA study (SRP183190) reported.

Recently, sequencing has advanced significantly from Sanger sequencing to next-generation sequencing (NGS). For example, whole-genome sequencing can produce comprehensive genomic information on a species^7^. Also, from NGS data, it is easier to obtain genetic information such as microsatellite markers, which are superior to other markers like RAPD and AFLP and are already used in other *Mangifera* species^8,9^. Microsatellite markers can determine distinct variations at the level of species as they are codominant; as a result, they are widely used in population and genetic studies^10^. Microsatellite markers have also been used to determine the genetic variation in *M. indica*^11^. Although microsatellites are important for taxonomy and the study of genetic conservation, no *M. casturi*-specific microsatellite markers have been reported.

In recent years, Sanger sequencing approaches have been utilized for DNA barcoding. DNA barcoding methods based on chloroplast regions, such as *rbcL*, *matK*^12^, and *trnH-psbA*^13^, internal transcribed spacers (ITS), and second internal transcribed spacers (ITS2) from nuclear ribosomal DNA^14^, have been widely used for phylogenetic analysis at various taxonomic levels. These DNA barcoding markers from chloroplast regions can also be determined at the genus or family level because of their inheritance from a maternal ancestor. On the other hand, the ITS region can determine the barcoding of the paternal and maternal ancestor. However, DNA barcoding sequences for *M. casturi* have not been recorded in any public database. As a result, there have been no phylogenetic studies of *M. casturi* using DNA barcoding to achieve accurate identification at the taxonomy level.

This study aimed to collect genomic information from *M. casturi* using NGS and Sanger and to analyze and determine the genetic variation among the *M. casturi* hybrids. Microsatellite markers were used to assess genetic variation among *M. casturi* hybrids, Kasturi, Cuban, Pelipisan, and Pinari. Furthermore, at a higher taxonomy level, phylogenetic analysis of *M. casturi hybrids* and Mangifera species was performed using DNA barcoding. In addition, there is no clear information and proof about the genetic variation and relationship among *M. casturi* hybrids. In this study, we propose a candidate ancestor from a natural hybridization of *M. indica* and *M. quadrifid*a.

## Results

In this study, 11.01 Gbp of *M. casturi* DNA was obtained with high-throughput sequencing using an Illumina HiSeq 4000 system with paired end 150 bp reads. The raw data were registered in the DDBJ with accession number DRA011022. Clean reads were obtained via filtering, and 10.95 Gbp of de novo genome assembly was performed using a Ray Assembler. We obtained 259 872 scaffolds with an N50 value of 1 445 bp and a maximum scaffold length of 144 601 bp (Table 1). The genome assembly and annotation completeness were assessed using BUSCO, and complete ratio, universal single-copy orthologs were found to be 42.3% similar using a plant reference database (Table 2).

**Table 1:** Statistics of de novo assembly from *M. casturi* using Ray Assembler.

Features	Number
Raw reads (bases)	73.44 million (11.01 Gbp)
Clean reads (bases)	73.10 million (10.95 Gbp)
Number of Scaffolds	259 872
N50	1 445 bp
Mean length	947.68 bp
Longest Scaffold	144 601 bp

**Table 2:** Summarized benchmarks in BUSCO annotation from *M. casturi* scaffolds.

No	Categories	Number	Ratio (%)
1	Complete and single-copy BUSCOs (S)	608	42.3
2	Complete and duplicated BUSCOs (D)	36	2.5
3	Fragmented BUSCOs (F)	241	16.7
4	Missing BUSCOs (M)	555	38.5

Microsatellite markers were identified using the MISA program, producing 11 040 sequences with at least one microsatellite motif, and 770 sequences with more than one site (Table 3). The trinucleotide motifs predominated with 52.77%, followed by the dinucleotide motif with 33.3%. Fourteen candidate sequences were selected and identified (Table 4). Finally, all the confirmed primers were amplified and registered in the DDBJ with accession numbers (Table 4).

**Table 3:** Number of microsatellite regions observed in *M. casturi* scaffolds, total and subdivided by motif.

Characteristics	Number
Total number of identified SSRs	11 040
Number of SSR containing contig	10 160
Contig containing more than 1 SSR	770
SSRs present in compound formation	272
**Motif**	
Dinucleotide	3 680
Trinucleotide	5 826
Tetranucleotide	1 194
Pentanucleotide	213
Hexanucleotide	102
Heptanucleotide	25

**Table 4:** Summary statistics of the fourteen analyzed microsatellite loci.

No	Locus	Primer sequence (5'-3')	Sequence Motif	Allele size range	NA	**H**_**o**_	**H**_**e**_	**F**_**ST**_	**I**	DDBJ accession
1	mc122955	F : TGTTGATGGTAAGGATTTGGTGT	(GGATG)6	168–178	2	0.50	0.50	0.60	0.69	LC594546
		R : TCAGGTGAGTATGTATTGTGCA								
2	mc148231	F : TCCCTCCCCTAAACCCTTCT	(ACCCTAA)5	188–209	4	0.68	0.68	0.76	1.24	LC594549
		R : GCTTCTCCTTGCCTCTAAATCCT								
3	mc151578	F : GAGCCTTGTACTCGTTCAATGA	(CAAGCT)8	273–279	5	0.25	0.78	0.68	1.56	LC594547
		R : ACGAGCTTAAAATGAGTTTGACT								
4	mc167596	F : AGCTGAACCTTGTTGCCCTT	(GA)27	192–224	3	0.16	0.40	0.58	0.72	LC594539
		R : TCTGCTTGTTGGAACTGAACA								
5	mc176197	F : TGTATGCCCGAATTGTTCCAAC	(AC)19	237–250	3	0.50	0.62	0.20	1.04	LC594537
		R : GCTGGCTTTAATGGAAGTTGCA								
6	mc211123	F : GGATGGTGGATGTCAGATTTTCG	(TGAAGT)6	323–339	6	0.60	0.76	0.21	1.60	LC594548
		R : CGAAGAGAACGGGTCCCTTG								
7	mc21672	F : TGGTTGGTAAGAAGTAGGATTC	(ATAC)11	263–264	4	0.00	0.61	1.00	1.15	LC594543
		R : CACAATGCAAATCACTCCTC								
8	mc230178	F : AGACAGCCATAATTTGCCCCA	(ATG)12	162–188	7	0.62	0.80	0.22	1.75	LC594541
		R : GCTGGAGGTTGATCAGGGTC								
9	mc28107	F : GGTGTGCGTTCTGTTTTGACA	(TG)28	211–250	5	0.00	0.78	1.00	1.55	LC594540
		R : CAGCAGCATCAACACAAGCA								
10	mc4673	F : TTTCCAAAGCCAAGACTCTC	(TAAACCC)5	231–245	3	0.25	0.66	0.62	1.08	LC594550
		R : AAAATTGTATTCATTAAGCCCCT								
11	mc58089	F : TCTTGTCGTCGAATCAAACTCA	(AT)22	264–287	7	0.38	0.76	0.51	1.66	LC594538
		R : CTCGGTCTATCAATGGTGTAGGT								
12	mc8693	F : CGAAGGGTTGAGGTTTGGGT	(CTTTT)7	159–183	4	0.62	0.68	0.08	1.22	LC594545
		R : AAAGAGTGAGAGGGTTGCGT								
13	mc88075	F : CTCCAATCGAACAACCCAGC	(TTA)15	278–286	3	0.00	0.57	1.00	0.96	LC594542
		R : AGGGGTGCATATGGAGGATT								
14	mc88387	F : CCATTTCGACGATGTTGGAAGT	(TATG)10	251–252	2	0.00	0.50	1.00	0.69	LC594544
		R : GCAACCCTTACCAACAAGCA								

*F* forward primer sequence, *R* reverse primer sequence, *NA* number of alleles, *H*_*o*_ observed heterozygosity, *H*_*e*_ expected heterozygosity, *F*_*ST*_ fixation index, *I* Shannon's information index.

Eight samples, namely Pelipisan, Cuban, Pinari, Kasturi, Rawa-rawa, *M. foetida*, *M. quadrifida*, and *M. indica* (Supplementary Table 1), were used to validate and determine the allele size of the microsatellite markers using QIAxcel capillary electrophoresis. The 14 primers produced 58 alleles in total and a mean of 4.14 alleles per locus (Table 4). All the loci were polymorphic (Table 5). The observed heterozygosity (H_o_) ranged from 0 for 4 markers (mc21672, mc28107, mc88075, and mc88387) to 0.62 for the mc8693 locus with a mean H_o_ of 0.26. The expected heterozygosity (H_e_) ranged from 0.40 for the mc167596 locus to 0.80 for the mc230178 locus, with a mean of 0.65. The fixation index (F_ST_), reflecting the degree of genetic differentiation, ranged from 0.08 to 1.00 with an average of 0.60 per locus. Lastly, Shannon’s information index ranged from 0.69 and 1.75 with a mean of 1.21.

**Table 5:** Allele size information of microsatellite loci.

No	Locus	Allele size (bp)
1	mc122955	168,178
2	mc148231	188,196,203,209
3	mc151578	256,270,273,279,284
4	mc167596	193,224,226
5	mc176197	235,237,249
6	mc211123	318,323,333,335,339, 340
7	mc21672	255,256,257,263
8	mc230178	162,164,170,173,178,185,188
9	mc28107	204,211,214,225,250
10	mc4673	231,238,246
11	mc58089	261,264,273,279,282,283,287
12	mc8693	157,160,167,182
13	mc88075	267,278,286
14	mc88387	251,253

The mc230178 and mc58089 loci produced seven alleles from eight samples, while the mc122955 and mc88387 loci produced two alleles. Some loci, namely mc176197, mc21672, and mc88075, displayed the same alleles between Kasturi and Cuban. In the mc88387 locus, only the Kasturi sample was not amplified; thus, this locus might have a null allele in Kasturi. Therefore, mc88387 locus can be used to identify *M. casturi* accessions in a natural population, as it is dissimilar to other *Mangifera* species.

**Figure 2:**
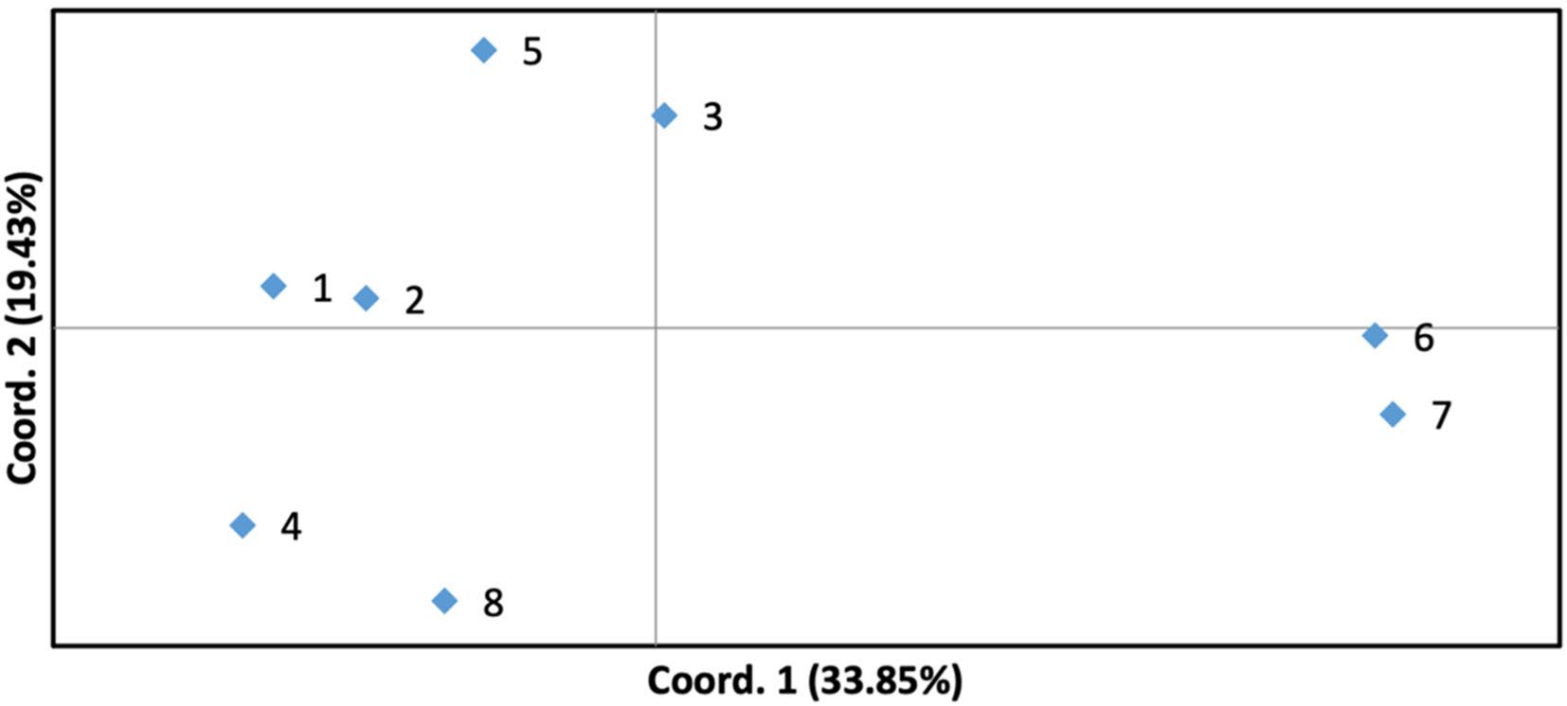
Principal Coordinates Analysis (PCoA) via covariance matrix with data standardization in four *M. casturi* hybrids and three closely related species using 14 microsatellite loci (1: Hambawang (*M. foetida*), 2: Pelipisan, 3: Cuban, 4: Pinari, 5: Kasturi, 6: *M. quadrifida*, 7: Rawa-rawa, 8: *M. indica*).

Furthermore, principal coordinate analysis was performed using the GENALEX 6.501, indicating that 33.85% of the variance within the microsatellite data was graphed by the first axis, and 19.43% by the second axis (Fig. 2). Additionally, the eight samples could clearly group into three clusters, Hambawang (*M. foetida*), Pelipisan, Pinari, *M. indica* in one cluster, while Kasturi and Cuban in the second cluster, and Rawa-rawa and *M. quadrifida* in the third cluster.

**Figure 3:**
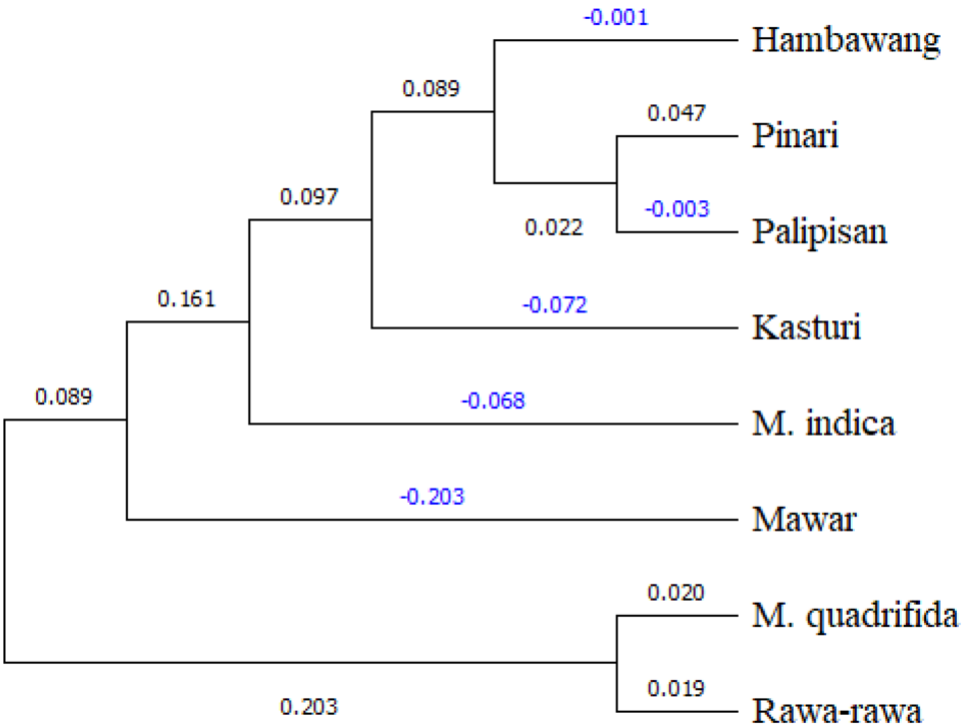
Dendrogram for UPGMA analysis in four *M. casturi* hybrids and four closely related species using 14 microsatellite loci.

The result was also presented in a UPGMA dendrogram (Fig. 3). *M. quadrifida* and Rawa-rawa were placed in the same clade. All the *M. casturi* accessions were in the same clade as *M. indica* and Hambawang (*M. foetida*). Cuban were most closely related to *M. indica*. However, Kasturi and Pelipisan were in the same clade where some loci showed similar alleles. Thus, these accessions had a closer genetic relationship to each other than to Cuban. However, Pinari also exhibited distinct genetic differences from the other *M. casturi* accessions, even though Cuban was quite distant from other *M. casturi* accessions.

**Figure 4:**
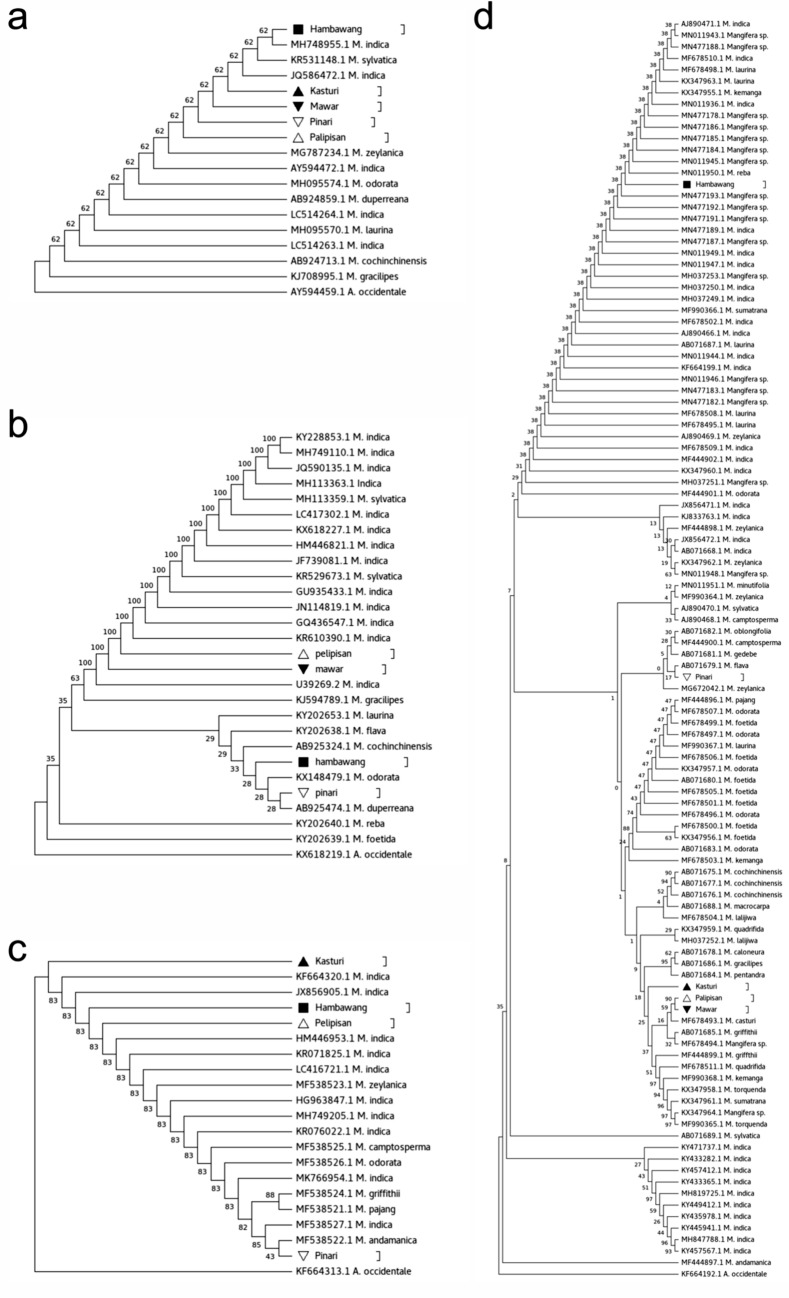
Phylogenetic analysis of *M. casturi* hybrids compared to the other Mangifera species (deposited in NCBI, Supplementary Table 2) using (**a)** *matK*, (**b**) *rbcL*, (**c**) *trnH-psbA*, and (**d**) internal transcribed spacers (ITS) by Maximum Likelihood method. (The evolutionary history was inferred by using the Maximum Likelihood method and Tamura 3-parameter model for *matK*, *trnH-psbA*, ITS and Jukes-Cantor model for *rbcL*. The bootstrap consensus tree was inferred from 10 000 replicates. The tree is rooted with the outgroup, *Anacardium occidentale.*).

Next, phylogenetic analysis was performed using three widely used chloroplast markers, *matK*, *rbcL*, and *trnH-psbA* (Fig. 4). The *matK*, *rbcL*, and *trnH-psbA* sequences of Kasturi, Cuban, Pelipisan, Pinari, and Hambawang were obtain using a 3500 Genetic Analyzer (Applied Biosystems) and were deposited in the DDBJ Nucleotide Sequence Submission System under the accession number LC602976- LC602993. However, the *matK*, *rbcL*, and *trnH-psbA* sequences from other Mangifera species were downloaded from the public nucleotide database of NCBI (Supplementary File 2). The *matK* phylogenetic tree showed that Kasturi, Cuban, Pelipisan, Pinari, and Hambawang belonged to one group with *M. indica* and *M. sylvatica*. In comparison, the *rbcL* phylogenetic tree placed Cuban and Pelipisan into the same clade as almost all *M. indica* accessions. Meanwhile, Pinari and Hambawang, separated from this clade, were joined with the *M. laurina, M. flava, M. cochinchinensis, M. odorata*, and *M. duperreana* clades. In contrast, the phylogenetic tree analysis using *trnH-psbA* placed Kasturi, Pelipisan, and Hambawang with *M. indica*. Lastly, Pinari was close to *M. odorata, M. griffithii, M. pajang, M. andamanica*, and *M. indica*.

On the other hand, the ITS phylogenetic tree produced three large groups: Indica 1, indica 2, and a group containing Kasturi, Cuban, Pelipisan, and Pinari. Hambawang was included in the indica 2 group. Meanwhile, Pinari was placed in a sub-group with *M. oblongifolia, M. camptosperma, M. gedebe,* and *M. flava*. Lastly, Kasturi, Cuban, and Pelipisan were included in the other sub-groups with *M. casturi* (MF678493.1), *M. griffthii, M. quadrifida, M. kemanga, M. torquenda,* and *M. sumatrana*.

## Discussion

The *Mangifera* genus originates from southeast Asia and has polyembryonic seeds, derived from gametes or nucellar cell components^2^. Most *Mangifera* flowers are either hermaphrodites or males^32^; thus, self-crossing can occur in various species. However, self-incompatibility in the *Mangifera* genus has been reported in several types of mangos^33^, suggesting that various *Mangifera* species can cross-hybridize^2,34^. As a result, cross-hybridization in the natural populations has produced many interspecies, including *M. odorata* (Kuini), a natural hybrid between *M. indica* and *M. foetida*^9^.

Microsatellite markers have been used successfully to determine genetic variation among many plants^35,36^. Microsatellites in Mangifera species that were previously identified in M. indica have been useful in the genetic analysis of genus Mangifera and its related genera^37,38^. In This study, genetic analysis has revealed markers in 14 microsatellite loci and that different allele sizes have arisen from four accessions of *M. casturi* namely Kasturi, Cuban, Pelipisan, and Pinari. The expected heterozygosity (He) value of the microsatellite markers used in the *M. casturi* analysis ranged between 0.40 and 0.80, with an average of 0.65, which indicated that the highly informative microsatellite markers could be employed in genetic diversity studies of *M. casturi*. In this study, a high level of genetic variation was discovered in *M. casturi* accessions, likely arising from repetitive interspecific hybridization. In Petunia, microsatellite markers have determined genetic differentiation and hybrid identification^39^. The accessions of Kasturi, Cuban, and Pelipisan were more closely related than Pinari. Kasturi and Cuban are very similar in fruit size. However, morphologically, the fruit shape of Kasturi is more oval than Cuban. In contrast, a Pelipisan fruit is more oval and slightly larger than Kasturi and Cuban. Pinari has the largest fruit size among the *M. casturi* accessions. Lastly, Pinari is classified into the *M. casturi* group by the locals, based on its purplish skin similar to that of other *M. casturi* accessions ^4^.

Intraspecies genetic variation can occur because of multiple cross-hybridizations among several species. Using microsatellite markers, hybridization between *Juglans regia* and *J. cathayensis* indicated a rare phenomenon and backcrosses between hybrids and either of the parental species^40^. In addition, Kuini (*M. odorata*), a natural hybrid between *M. indica* and *M. foetida* was revealed by AFLP analysis to represent a simultaneous backcross between the F1 hybrids of Kuini and *M. foetida*^4,9^. On the other hand, SNP analysis using double-digest restriction-site-associated DNA (ddRAD)^4^, revealed *M. casturi* to be a natural hybrid between *M. indica* and *M. quadrifida*, whereas their F1 hybrid was a backcross with *M. indica*. Morphologically, *M. casturi* is very close to *M. quadrifida*, with the same purplish skin and small fruit size^4^. Therefore, *M. casturi* has several types known as Kasturi, Cuban, Pelipisan, and Pinari. These hybrids are believed to be hybrids between *M. indica* and *M. quadrifida* and backcrosses between hybrids and either of the ancestors.

In an allopolyploid plant such as mangosteen (*Garcinia mangostana*), microsatellite markers indicate cross-hybridization with multiple ancestors, including *G. malaccensis*, *G. celebica*, and *G. porrecta*^22^. In this study, microsatellite analysis results showed that four accessions of *M. casturi,* namely Kasturi, Cuban, Pelipisan, and Pinari had allelic differences in all the microsatellite loci. However, allele sharing between the four accessions was detected in the mc8693 locus with an allele size of 160/182, indicating that these accessions were derived from the same ancestor, *M. indica*. In contrast, the allele differences in the microsatellite loci suggested that the four *M. casturi* accessions underwent cross-hybridization with multiple ancestors. In Oaks (*Quercus* spp.), microsatellites indicate sharing most alleles in their hybrids than recurrent gene flow^41^.

Plant taxonomists have used the chloroplast coding regions *matK*, *rbcL*, and *trnH-psbA* intergenic spacers in DNA barcoding analysis^42^. DNA barcoding analysis using *matK* and *rbcL* implied very high nucleotide conservation between the four *M. casturi* accessions. Also, this evidence indicated that the maternal ancestor of these accessions was identical and that *M. indica* was one of their maternal ancestors. Additional evidence was found in the *trnH-psbA* phylogenetic tree, where Pinari had a different maternal ancestor from the other accessions. Therefore, one of the *M. casturi* hybrids may have crossed with other Mangifera species as the maternal ancestor.

In addition to chloroplast markers, the nuclear ribosomal internal transcribed spacer (ITS) region has also been indicated as a barcoding region^43^. ITS sequences are highly variable, conserved region, and biparentally inherited in most angiosperms and widely used to construct a phylogenetic tree for inferring the hybrid origin of species^44–47^. The ITS phylogenetic tree also revealed that three accessions of *M. casturi*, excluding Pinari, belonged to the same sub-group, in contrast to a previous hypothesis that *M. casturi* was a cross-hybrid between *M. indica* and *M. quadrifida*^4^. Lastly, the DNA barcoding results also supported the hypothesis that the F1 hybrids of *M. casturi* crossed with other *Mangifera* produce natural hybrids of *M. casturi* that had a high level of genetic variation.

In this study, the genomic data revealed the genetic variation and ancestral origin of *M. casturi* hybrids. Based on genomic data, we have identified that *M. casturi*, which is endemic to Kalimantan Selatan, consists of 4 types, namely Kasturi, Cuban, Pelipisan, and Pinari. In Addition, based on the combination of microsatellite data, and DNA barcoding, *M. casturi* hybrids are natural hybrids between *M. indica* and *M. quadrifida*. Moreover, the genomic data represent an important genetic resource for breeding and improving the characteristics of this local mango in the future. The habitat of *M. casturi* is severely threatened; as a result, it is classified as extinct in the wild. Thus, more intensive conservation efforts are necessary. Moreover, since *M. casturi* varieties have never been confirmed or registered by authorities, the results of this study can help breeders and the local government to officially document this local mango and one of their elite germplasm.

## Methods

*M. casturi* accessions were collected from the Banjar district, Kalimantan Selatan, in the southern region of Kalimantan, three Mangifera species were collected from Banua Botanical Garden (*M. quadrifida*, *M. foetida*) and Dramaga, Bogor, West Java (*M. indica*) (Fig. 1; Supplementary Table 1). To analyze whole genome sequencing, genomic DNA was isolated from Kasturi accession using a DNeasy Power Plant kit (Qiagen) following the manufacturer's protocol. The quality and quantity of DNA were analyzed using a NanoPhotometer NP80 Touch (Implen) spectrophotometer. Genomic DNA samples were sent to Novogen-AIT Singapore with 150 paired-end (PE) reads collected using an Illumina HiSeq4000 system. Raw reads were quality controlled using FASTQC Version 0.11.9 (https://www.bioinformatics.babraham.ac.uk/projects/fastqc/). ^15^, and clean reads were filtered using the Fastp version 0.20.1 (https://github.com/OpenGene/fastp) with default parameters^16^. Clean reads were assembled using a Ray version 2.1.0 (https://github.com/sebhtml/ray) with default parameters (-k 63 -minimum-contig-length 200)^17^ under the Maser Platform (https://cell-innovation.nig.ac.jp/). ^18^. The assessment of genome assembly was performed to check the assembled contig quality using BUSCO version 3.0.2 (https://busco.ezlab.org). ^19^ on the Maser Platform with default parameters.

### Microsatellite marker development and validation

Microsatellite markers were extracted using the MISA version 2.1 (https://webblast.ipk-gatersleben.de/misa/). ^20^ from *M. casturi* scaffolds, the parameters being set to the following minimum repeat levels: six for two bases, and five for three, four, five, and six bases. The difference between microsatellite motifs was 100 bases. The microsatellite motif-containing sequences were selected based on parameters; (1) the flanking region are at least 150 bp long in both directions (2) microsatellite repeats have the longest repeat motifs. The primer was designed using the web version of Primer 3 with default parameters^21^.

Genomic DNA was isolated using the modified CTAB method, with a slight modification^22^. The quality and quantity of DNA were assessed using a NanoPhotometer NP80 Touch (Implen). A Type-it microsatellite PCR kit (Qiagen) was used to analyze the microsatellite markers. PCR master mix was prepared with a mixture of 3.2 μL RNase-free water, 5 μL 2 × Type-it Multiplex PCR Master Mix, 0.4 Q solution, 0.2 μL of 10 μM forward primer, and 0.2 μL of 10 μM reverse primer. PCR was performed using a SimpliAMP Thermo Cycler (Applied Biosystems). The PCR conditions were as follows: initial conditions of PCR pre-denaturation at 95 °C for 5 min, followed by 32 cycles of denaturation at 95 °C for 30 s, annealing at 57 °C for 1 min 30 s, extension at 70 °C for 30 s, and final extension at 60 °C for 30 min. The amplicons were checked using 1% electrophoresis gel in TAE buffer for 20 min at 100 v. Before loading the sample into QIAxcel capillary electrophoresis (Qiagen), the sample was diluted twice and then run using a QIAxcel DNA High Resolution Kit (Qiagen). Allele size data were confirmed and processed manually using QIAxcel ScreenGel version 1.4.0 (https://www.qiagen.com/us/products/instruments-and-automation/quality-control-fragment-analysis/qiaxcel-advanced-system/?catno=9021163).

Descriptive statistics were calculated using GENALEX version 6.501 (https://biology-assets.anu.edu.au/GenAlEx/) for each microsatellite marker, including the number of alleles (Na) per locus, and both observed (Ho), and expected (He) heterozygosity, fixation index (F_ST_), and Shannon's information index (I). The principal coordinate analysis (PCoA) via Covariance matrix with data standardization was also performed using GENALEX 6.501. The microsatellite data were processed using the Phylip version 3.695 (https://evolution.genetics.washington.edu/phylip.html) with the unweighted pair group method and arithmetic mean (UPGMA) method. The resulting dendrogram was edited using the program MEGA-X Software (https://www.megasoftware.net)^23^.

### DNA barcoding and phylogenetic analysis

For the DNA barcoding analysis, we used three chloroplast genes: *matK*^24^, *rbcL*^25^, and *trnH-psbA*^26^ and one nuclear DNA region of the internal transcribed spacer (ITS)^27^. PCR barcoding was performed using KOD Plus (Toyobo) according to the manufacturer's protocol. The PCR products were cleaned using ExoSAP-IT PCR Product Cleanup Reagent (Applied Biosystems). Then, PCR sequencing was carried out with a BigDye Terminator v3.1 Cycle Sequencing Kit (Applied Biosystems), followed by purification using a BigDye XTerminator Purification Kit (Applied Biosystems) according to the manufacturer protocol. The sequencing products were performed using a 3500 Genetic Analyzer (Applied Biosystems). Sequence data were analyzed using Sequencing Analysis Software version 6.0 (https://www.thermofisher.com/order/catalog/product/4474950), and the data were processed with ATGC-MAC version 7 (https://www.genetyx.co.jp) and MEGA-X software (https://www.megasoftware.net). ^23^.

Phylogenetic trees were inferred using the maximum likelihood method and constructed using MEGA-X software^28^. The Mangifera sequences of *matK*, *rbcL*, and *trnH-psbA*, and ITS complete sequences were downloaded from NCBI (Supplementary Table 2). The best DNA model was calculated using MEGA X for each marker^29,30^. Phylogenetic trees were tested using 10 000 bootstrap replicates^31^.

### Ethics approval and consent to participate

All experiments were performed in accordance with relevant guidelines and regulations. The experimental research has complied with Bogor Agricultural University research regulation (No: 11/SA-IPB/P/2016 on research and publication ethics), and the field study was in accordance with the national legislations of Indonesian Law Number 5/1990 on biological diversity conservation and Indonesia Law Number 11/2013 on the ratification of the Nagoya Protocol. *M. casturi* samples were collected and exported from Banjarbaru, South Kalimantan to Bogor, West Java with the permission (No: 2020.2.1702.0.K12.000044) from Plant Quarantine Division of National Agency for Agricultural Quarantine in Banjarbaru, South Kalimantan following permit approvals from South Kalimantan Natural Resources Conservation Agency/BKSDA of the Ministry of Environment and Forestry of the Republic of Indonesia (KLHK) as agency in charge of managing conservation areas including protected plant in the territory, particularly the nature reserve forests (wildlife, nature reserves) and national park. The *M. casturi* samples from Banjar, South Kalimantan as herbaria voucher (received by Agung Sriyono) were duplicated and stored in Banua Botanical Garden, Province of South Kalimantan, Banjarbaru, Indonesia.

## Supplementary Information

Supplementary Information 1.

Supplementary Information 2.

## Data Availability

All sequence data from the next generation sequencing during the current study have been submitted to the DDBJ Read Archive (DRA) under the BioProject accession number PRJDB10715: http://trace.ddbj.nig.ac.jp/BPSearch/bioproject?acc=PRJDB10715. All sequence data from DNA barcoding analysis during the current study have been submitted to the DDBJ Nucleotide Sequence Submission System under the accession number of LC602976- LC602993.

## References

[1] Rhodes, L., Maxted, N. Mangifera, C. The IUCN red list of threatened species. 2016.

[2] Kostermans AJGH, Bompard JM (1993). The Mangoes: Their Botany, Nomenclature, Horticulture, and Utilization.

[3] Fitmawati F, Hayati I, Mahatma R, Suzanti F (2018). Phylogenetic Study of Mangifera from Sumatra, Indonesia using Nuclear and Chloroplast DNA Sequences. Sabrao J. Breed Genet..

[4] Warschefsky, E. The evolution and domestication genetics of the mango genus, mangifera (Anacardiaceae). Doctoral dissertation. Florida International University; 2018.

[5] Iyer CPA, Schnell RJ, Mango T (2009). Breeding and genetics. Botany, Production and Uses.

[6] Suhartono E, Viani E, Rahmadhan M, Gultom I, Rakhman M, Indrawardhana D (2012). Total flavonoid and antioxidant activity of some selected medicinal plants in South Kalimantan of Indonesian. APCBEE Proc..

[7] Ekblom R, Galindo J (2011). Applications of next generation sequencing in molecular ecology of non-model organisms. Heredity (Edinb)..

[8] Eiadthong W, Yonemori K, Kanzaki S, Sugiura A, Utsunomiya N, Subhadrabandhu S (2000). Amplified fragment length polymorphism analysis for studying genetic relationships among Mangifera species in Thailand. J. Am. Soc. Hortic. Sci..

[9] Teo LL, Kiew R, Set O, Lee SK, Gan YY (2002). Hybrid status of kuwini, Mangifera odorata Griff (Anacardiaceae) verified by amplified fragment length polymorphism. Mol. Ecol..

[10] Zane L, Bargelloni L, Patarnello T (2002). Strategies for microsatellite isolation: A review. Mol. Ecol..

[11] Viruel MA, Escribano P, Barbieri M, Ferri M, Hormaza JI (2005). Fingerprinting, embryo type and geographic differentiation in mango (Mangifera indica L, Anacardiaceae) with microsatellites. Mol. Breed..

[12] Hollingsworth PM, Forrest LL, Spouge JL, Hajibabaei M, Ratnasingham S, van der Bank M (2009). A DNA barcode for land plants. Proc. Natl. Acad. Sci..

[13] Pang X, Liu C, Shi L, Liu R, Liang D, Li H (2012). Utility of the trnH–psbA intergenic spacer region and its combinations as plant DNA barcodes: A meta-analysis. PLoS ONE.

[14] Li D-Z, Gao L-M, Li H-T, Wang H, Ge X-J, Liu J-Q (2011). Comparative analysis of a large dataset indicates that internal transcribed spacer (ITS) should be incorporated into the core barcode for seed plants. Proc. Natl. Acad. Sci..

[15] Andrews S. FastQC a quality-control tool for high-throughput sequence data. 2010. http://www.bioinformatics.babraham.ac.uk/projects/fastqc/.

[16] Chen S, Zhou Y, Chen Y, Gu J (2018). fastp: An ultra-fast all-in-one FASTQ preprocessor. Bioinformatics.

[17] Boisvert S, Laviolette F, Corbeil J (2010). Ray: Simultaneous assembly of reads from a mix of high-throughput sequencing technologies. J. Comput. Biol..

[18] Kinjo S, Monma N, Misu S, Kitamura N, Imoto J, Yoshitake K (2018). Maser: One-stop platform for NGS big data from analysis to visualization. Database.

[19] Simão FA, Waterhouse RM, Ioannidis P, Kriventseva EV, Zdobnov EM (2015). BUSCO: Assessing genome assembly and annotation completeness with single-copy orthologs. Bioinformatics.

[20] Thiel T, Michalek W, Varshney R, Graner A (2003). Exploiting EST databases for the development and characterization of gene-derived SSR-markers in barley (Hordeum vulgare L.). Theor. Appl. Genet..

[21] Untergasser A, Cutcutache I, Koressaar T, Ye J, Faircloth BC, Remm M (2012). Primer3—new capabilities and interfaces. Nucleic Acids Res..

[22] Matra DD, Poerwanto R, Santosa E, Sobir HH, Anzai H (2016). Analysis of allelic diversity and genetic relationships among cultivated mangosteen (*Garcinia mangostana* L.) in Java, Indonesia using microsatellite markers and morphological characters. Trop. Plant Biol..

[23] Stecher G, Tamura K, Kumar S (2020). Molecular evolutionary genetics analysis (MEGA) for macOS. Mol. Biol. Evol..

[24] Cuénoud P, Savolainen V, Chatrou LW, Powell M, Grayer RJ, Chase MW (2002). Molecular phylogenetics of Caryophyllales based on nuclear 18S rDNA and plastid rbcL, atpB, and matK DNA sequences. Am. J. Bot..

[25] Kress WJ, Wurdack KJ, Zimmer EA, Weigt LA, Janzen DH (2005). Use of DNA barcodes to identify flowering plants. Proc. Natl. Acad. Sci. U.S.A..

[26] Sang T, Crawford D, Stuessy T, Sang T, Crawford DJ, Stuessy TF (1997). Chloroplast DNA phylogeny, reticulate evolution, and biogeography of Paeonia (Paeoniaceae). Am. J. Bot..

[27] Cheng T, Xu C, Lei L, Li C, Zhang Y, Zhou S (2016). Barcoding the kingdom Plantae: new PCR primers for ITS regions of plants with improved universality and specificity. Mol. Ecol. Resour..

[28] Kumar S, Stecher G, Li M, Knyaz C, Tamura K (2018). MEGA X: Molecular evolutionary genetics analysis across computing platforms. Mol. Biol. Evol..

[29] Jukes TH, Cantor CR, Munro HN (1969). Evolution of protein molecules. Mammalian Protein Metabolism.

[30] Tamura K (1992). Estimation of the number of nucleotide substitutions when there are strong transition-transversion and G+C-content biases. Mol. Biol. Evol..

[31] Felsenstein J (1985). Confidence limits on phylogenies: An approach using the bootstrap. Evolution.

[32] Ledesma N, Campbell RJ, Poor HW, Figueroa JJ, Zona S (2017). Floral morphology of seven Mangifera species. Acta Hortic..

[33] Dutta SK, Srivastav M, Rymbai H, Chaudhary R, Singh AK, Dubey AK (2013). Pollen–pistil interaction studies in mango (Mangifera indica L.) cultivars. Sci. Hortic..

[34] Mukherjee SK, Litz RE, Litz RE (2009). Introduction: Botany and Importance. The Mango: Botany, production and uses.

[35] Dirlewanger E, Cosson P, Tavaud M, Aranzana M, Poizat C, Zanetto A (2002). Development of microsatellite markers in peach Prunus persica (L.) Batsch and their use in genetic diversity analysis in peach and sweet cherry (Prunus avium L.). Theor. Appl. Genet..

[36] Emanuelli F, Lorenzi S, Grzeskowiak L, Catalano V, Stefanini M, Troggio M (2013). Genetic diversity and population structure assessed by SSR and SNP markers in a large germplasm collection of grape. BMC Plant Biol..

[37] Schnell RJ, Olano CT, Quintanilla WE, Meerow AW (2005). Isolation and characterization of 15 microsatellite loci from mango (Mangifera indica L.) and cross-species amplification in closely related taxa. Mol. Ecol. Notes..

[38] Ravishankar KV, Mani BH, Anand L, Dinesh MR (2011). Development of new microsatellite markers from Mango (*Mangifera indica*) and cross-species amplification. Am. J. Bot..

[39] Turchetto C, Segatto AL, Beduschi J, Bonatto SL, Freitas LB (2015). Genetic differentiation and hybrid identification using microsatellite markers in closely related wild species. AoB Plants..

[40] Shu Z, Zhang X, Yu D, Xue S, Wang H (2016). Natural hybridization between Persian Walnut and Chinese Walnut revealed by simple sequence repeat markers. J. Am. Soc. Hort. Sci..

[41] Muir G, Schlötterer C (2005). Evidence for shared ancestral polymorphism rather than recurrent gene flow at microsatellite loci differentiating two hybridizing oaks (Quercus spp.). Mol. Ecol..

[42] Pang X, Liu C, Shi L, Liu R, Liang D, Li H (2012). Utility of the trnH–psbA intergenic spacer region and its combinations as plant DNA barcodes: a meta-analysis. PLoS ONE.

[43] Kress WJ, Wurdack KJ, Zimmer EA, Weigt LA, Janzen DH (2005). Use of DNA barcodes to identify flowering plants. Proc. Natl. Acad. Sci. USA.

[44] Siripun KC, Schilling EE (2006). Molecular confirmation of the hybrid origin of *Eupatorium godfreyanum* (Asteraceae). Am. J. Bot..

[45] Álvarez I, Wendel JF (2003). Ribosomal ITS sequences and plant phylogenetic inference. Mol. Phylogenet. Evol..

[46] Sang T, Crawford DJ, Stuessy TF (1995). Documentation of reticulate evolution in peonies (paeonia) using internal transcribed spacer sequences of nuclear ribosomal DNA: Implications for biogeography and concerted evolution. Proc. Natl. Acad. Sci. USA.

[47] Baldwin BG (1992). Phylogenetic utility of the internal transcribed spacers of nuclear ribosomal DNA in plants: An example from the compositae. Mol. Phylogenet. Evol..

